# Review of Terms and Definitions Used in Descriptions of Running Shoes

**DOI:** 10.3390/ijerph17103562

**Published:** 2020-05-19

**Authors:** Ana Marchena-Rodriguez, Ana Belen Ortega-Avila, Pablo Cervera-Garvi, David Cabello-Manrique, Gabriel Gijon-Nogueron

**Affiliations:** 1 Department of Nursing and Podiatry, Faculty of Health Sciences, University of Malaga, Arquitecto Francisco Penalosa 3, Ampliación de Campus de Teatinos, 29071 Malaga, Spain; amarchena@uma.es (A.M.-R.); pcervera@uma.es (P.C.-G.); gagijon@uma.es (G.G.-N.); 2 Department of Physical Education and Sports, Faculty of Sports Sciences, University of Granada, 18071 Granada, Spain; dcabello@ugr.es; 3 Instituto de Investigación Biomédica de Málaga (IBIMA), 29010 Malaga, Spain

**Keywords:** footwear, sports shoes, running shoes, conventional running shoes, minimalist running shoes, barefoot running, motion control shoes

## Abstract

Objective: Our study aim is to identify and describe the definitions used for different types of running shoes. In addition, we highlight the existence of gaps in these concepts and propose possible new approaches. Methods: This review was undertaken in line with the guidelines proposed by Green et al., based on a literature search (until December 2019) of the PubMed, Web of Science, Scopus, SPORTDiscus and Google Scholar databases. A total of 23 papers met the inclusion criteria applied to identify the definition of running shoes. Results: Although there is a certain consensus on the characteristics of minimalist footwear, it is also described by other terms, such as barefoot-style or barefoot-simulating. Diverse terms are also used to describe other types of footwear, and in these cases, there is little or no consensus regarding their characteristics. Conclusions: The terms barefoot-simulated footwear, barefoot-style footwear, lightweight shoes and full minimalist shoes are all used to describe minimalist footwear. The expressions partial minimalist, uncushioned minimalist and transition shoes are used to describe footwear with non-consensual characteristics. Finally, labels such as shod shoes, standard cushioned running shoes, modern shoes, neutral protective running shoes, conventional, standardised, stability style or motion control shoes span a large group of footwear styles presenting different properties.

## 1. Introduction

As a sports discipline, running has been widely studied in terms of injuries, economy of movement, health improvements, etc. [1,2,3], and the footwear needed for this activity has evolved at a dramatic rate [4]. 

Currently, most runners choose to wear shoes, but despite the development of shoes aimed at improving performance and reducing injuries, in recent years the interest in running barefoot [5] or minimally shod [6] has increased considerably.

Many studies have been conducted to determine how barefoot, minimalist or standard running affects parameters such as biomechanics and overload [7,8], but in referring to these aspects of running, it is not always clear what characteristics of the footwear are assumed to be present in each case and what nomenclature should be employed.

Nevertheless, the characteristics of footwear termed “minimalist” are basically accepted among experts. Such shoes are those presenting minimal interference with the natural movement of the foot, providing great flexibility, low drop, light weight and minimal thickness of the sole, together with an absence of movement and stability control mechanisms [9].

On the other hand, various definitions have been offered of the three main types of running shoes, Barefoot, Minimalist and Standard [9,10,11]. Thus, in the grey literature, the term “barefoot shoes” has been used to refer to minimalist or “intermediate or natural” footwear. However, many professionals in this field who take their cue from grey literature or who contribute to it have created their own classifications, thus generating considerable confusion when this issue is discussed with runners and podiatric patients. Diverse approaches have also been taken in the scientific literature. Although the concept of minimalist shoes is agreed among experts, discrepancies continue to arise in defining different types of footwear. Thus, Young et al. [12] talked about “natural” running (forefoot striking), without specifying the footwear used this technique, and Cochrum [13] referred to “barefoot-style” footwear when he was really describing minimalist footwear. Lieberman queried that many minimal shoes were being advertised as barefoot shoes, and posed the question: how can running in a shoe, however minimal, be described as “barefoot”? [14].

A similarly wide range of expressions have been used in the scientific literature to describe “normal” running footwear, including standard cushioned running shoes [13], neutral style shoes [15], conventional shoes [16] or traditional running shoes [17].

In the absence of consensus or scientific reports determining an unequivocal classification of the footwear analysed in research studies (except that concerning minimalist footwear), some authors have focused on its technical characteristics, such as the out sole or mid sole material, or the control system [18,19].

In view of these considerations, our study aim is to identify and describe the definitions used for different types of running shoes, to highlight any gaps observed in these concepts and to propose possible new approaches.

## 2. Materials and Methods 

This narrative review was conducted in line with the guidelines proposed by Green et al. [20]. 

A computer-based literature search was performed of the following databases, from their indexation until April 2019: PubMed, Web of Science, Scopus, SPORTDiscus and Google Scholar. The search terms were “sports shoes”, “running shoes”, “conventional running shoes”, “minimalist running shoes”, “barefoot running” and ““shod running shoes”, with the connectors “and” “in”, “for”. Full publications and abstracts were screened and all relevant papers retrieved by two researchers, working independently.

The following inclusion criteria were applied: (1) The paper should include the name of a type of footwear within the sports discipline of running; (2) It should describe the characteristics of the footwear (3) The language of publication should be English, regardless of the country in which the study was conducted.

The exclusion criteria were: (1) Studies in which the sports shoe studied was identified by brand and model but not by type, according to the classifications currently used in the literature; (2) Papers that analysed biomechanics and running techniques. 

In the first stage of the review, a double-blinded assessment of titles and abstracts was carried out by two reviewers, working independently, to determine whether each item met the requirements for inclusion. In case of doubt, the full text of the article was evaluated (Annexe 1). References were exported and duplicate articles removed using reference management software (Mendeley Desktop v 1.19.4, London, UK).

## 3. Results

In total, 25 papers met the inclusion criteria. However, two of these were eliminated as we were unable to locate the full text or contact the author(s). Table 1 summarises the 23 studies analysed, following application of the inclusion and exclusion criteria, to identify the definitions made of running shoes.

## 4. Discussion

The aim of this study is to examine the different definitions of running shoes used in scientific literature on this subject. Some authors use the expression “barefoot-simulated” to refer to minimalist footwear, and “shod footwear” for the standard type [21,39].

The use of the “barefoot-simulated footwear” concept is associated with a running technique based on a more natural forefoot strike pattern, while shod footwear is associated with a standard running technique (shod running); approximately 90% of shod runners land on their heels [32].

Cochrum et al. used the term “barefoot-style footwear” to refer to the “five-toed minimal” footwear that would be classified as minimalist footwear according to the consensus of the minimalist index [13], which would also include the above-mentioned term “barefoot simulated footwear”. However, Fredericks et al. (2015) spoke about “barefoot without shoes” to refer to running barefoot [25]. The confusion is compounded by Giuliani et al. [39], who used the term “barefoot-simulating footwear” to refer to minimalist footwear and not to the pure concept of what is normally understood as barefoot.

Rixe at al. (2012) referred to “barefoot” in a minimalist debate and compared it with biomechanics, with shod shoes, but did not stipulate the characteristics associated with this type of footwear [40]. Therefore, their conclusions cannot be analysed according to our study criteria. Rothschild also discussed barefoot and minimalist shod running as one and the same category, and evaluated them globally within the same survey, in an approach that might be subject to interpretation bias. However, in the discussion section of this study, the authors stated that the two terms were not equivalent and that in the literature the term “minimalist shoe” was not specifically defined [24]. This question was later resolved with the Esculier consensus [9], which was adopted by Fuller (2019), who applied the minimalist index test to both minimalist and standard footwear, to determine the degree of minimalism present in each case. This test showed that the “minimalist” shoes considered scored 72% in the minimalist test, versus 12% for the standard shoes [16].

According to Grier et al., minimalist running shoes can be classified into three subgroups: barefoot style, minimalist and transition. These categories differ in the amount of cushioning provided and in the degree of heel-toe differential, which ranges from 0 to 9 mm [17]. In a related study, Murphy (2013) described “minimalist footwear” as that which maintains the freedom and essence of barefoot running without the cushioned midsole of standard running shoes [30].

Hollander et al. (2015) defined two types of minimalist footwear: cushioned and uncushioned. However, these extremes are too different to be both categorised as minimalist merely due to the low drop presented. In this respect, Ryan [26] and Hollander [31] both examined the same brand and model of running shoe, but in the first case it was termed “partial minimalist” and in the second, “cushioned minimalist”, which again highlights the lack of homogeneity in the terms used.

In 2015, efforts were made to reach a consensus on the characteristics and description of minimalist footwear. It would be helpful if in future research these consensus findings were applied [9]. However, in 2018, Moody et al. [35], described as minimalist footwear that which contained little or no drop, but which might or might not contain cushioning and/or motion control materials.

Another term appeared in 2016, when Da Silva Azevedo et al. used the term “transition shoes”, to describe a shoe with 12 mm drop, cushioning and control elements in the midfoot sole [37].

Murphy used the terms “modern running shoes” and “standard running shoes” for those in which the cushioning is typically made of foam (or other compliant material) and elevates the heel by 8–16 mm [30]. The footwear described in a recent paper by Kulmala et al. would fall within this classification, since the model examined (Brooks Ghost 6) had a heel-toe drop of 12 mm. Nevertheless, these authors used a different term, “Conventional cushioned running shoes” [41].

Knapik (2009) reported that the terms “motion control”, “stability” or “cushioned” shoes were applied interchangeably to 17 of the 19 shoes studied by the three entities consulted (store, manufacturer and magazine) [28]. In this respect, Grier et al., divided “traditional running shoes” into three subgroups: stability, cushioning and motion control shoes [17]. This discrepancy again reflects the lack of agreed criteria in the literature.

In another approach, Sayer et al. differentiated standard footwear into stability shoes and neutral shoes. The former provides pronation control and a considerable drop, of 13 mm (in this study, the Asics Kayano shoe was examined). The neutral shoes also had a large drop (10 mm) but lacked pronation control (the shoe analysed was the Asics Zacara 3). These two shoes were compared with the barefoot style. However, anti-pronator or neutral shoes, should be employed according to the user’s needs, not according to the characteristics integral to the shoe, such as a certain degree of knee flexion, which the authors attribute to the shoe itself. In fact, this study concluded that there was no difference in the moment of maximum knee flexion between the two types of footwear (stability and neutral), while the barefoot category presented a marked difference in this respect [15].

Langley et al. [27] propose yet another classification, identifying three subtypes within the “conventional” running shoe category: motion control, neutral and cushioned. According to these authors, motion control running shoes are designed to reduce the magnitude and/or rate of pronation with a view to enhancing the propulsive efficiency of the foot, in comparison to neutral and cushioned shoes. In contrast, cushioned running shoes aim to reduce the magnitude and/or rate of impact loading, and increase foot motion relative to neutral and motion control running shoes. Finally, neutral running shoes combine various motion control and cushioning features, seeking to achieve greater stability than with cushioned running shoes, and greater force attenuation than with motion control running shoes. In other studies, the concept of “cushioned running shoes” is intrinsic within that of conventional running shoes.

The extreme development of running shoe design was defined by Pollard et al., who introduced the term “maximum footwear”, a type that is currently very popular, providing extra cushioning of the entire midsole, from rearfoot to forefoot, but without any increase in the drop. This maximum footwear, therefore, could be viewed as forming part of traditional footwear, for heel-strikers and with a conventional degree of drop but with extra cushioning [32].

Ramsey et al. (2009) conducted a systematic review to evaluate footwear characteristics and the assessment methods used in studies of running-related injuries. This review showed that running shoes are described in many ways, with different terms sometimes referring to the same type of footwear [42]. Thus, Cauthon et al. [43] referred to “conventional running shoes” with respect to the same footwear described elsewhere as “standard or traditional”.

In 2013, Ryan et al. [26] studied “neutral” footwear and referred to “partial minimalist” and “total minimalist”. “Neutral” in this context was applied to the 99 study participants who obtained a pronounced, supine or neutral score on the Foot Posture Index (thus, strongly pronated or supinated participants were excluded). In other studies, such a shoe has been termed “conventional”, “standard” or “traditional.” When these authors refer to “partial minimalist” footwear, they mean that with a 4mm drop but containing control elements, while “total minimalist” footwear is that which has a 0 mm drop and no control elements.

With respect to the amount of the drop in the shoe, both partial and total minimalist shoes would be classed as minimalist footwear, but with different amounts of drop. In other words, application of the minimalist Esculier index [9] to these shoes would classify them both as minimalist in terms of drop, although the shoe with 4 mm drop would be considered “less minimalist”. However, the inclusion of control elements would exclude the shoe from being considered as minimalist footwear.

The above review of the literature reflects the great diversity of concepts regarding the terminology used for running shoes. The fact that different terms are sometimes used for shoes presenting the same characteristics, together with the diverse approaches adopted in the grey literature, means that confusion is often provoked. Even after the publication of the Esculier consensus and the European Running shoes categorization: A0 Barefoot, A1 Super light (shoes lower than 250 gr) body weight lower than 70 kg, A2 Intermediate (shoes lower than 300 gr) body weight lower than 75 kg, A3 Neutral (shoes 300–400 gr) body weight lower than 80 kg, A4 Stability (shoes 350–450 gr) body weight upper than 80 kg, A5 Trial running (shoes 300–450 gr), A6 Jogging, A7 Spike Shoe (shoes lower than 200 gr) for faster runners (sprinter 50 < 800m and middle distance 3000m in the track and field) [44,45,46], there remains considerable heterogeneity among research studies in this area, which makes it difficult to compare the results reported.

In view of these considerations, the aim of the present study is to integrate the different terms currently employed to define the types of running footwear used, thus facilitating greater clarity regarding the terms and the characteristics reflected in each case, and achieving a meaningful advance in the definition of running shoes.

## 5. Conclusions

The terms barefoot-simulated footwear, barefoot-style footwear, lightweight shoes and full minimalist shoes are all used to describe minimalist footwear. The expressions partial minimalist, uncushioned minimalist and transition shoes are used to describe footwear with non-consensual characteristics. Finally, labels such as shod shoes, standard cushioned running shoes, modern shoes, neutral protective running shoes, conventional, standardised, stability style or motion control shoes span a large group of footwear styles presenting different properties. 

This literature review of definitions of running shoes reflects the current situation in this field, highlighting the considerable variety observed and the continuing absence of consensus regarding terms such as transition, standard and barefoot. This situation provokes confusion in communications between researchers and among professionals and/or podiatric patients.

Further standardisation is needed of this terminology and of the definitions employed in each case, to enable quality research to be conducted into the use of different concepts and terms, and to facilitate systematic reviews to generate more evidence in this area.

## Figures and Tables

**Table 1:** Summary of the studies examined to extract the terms used for running shoes.

Author	Year	Term Used	Characteristics of the Concept	Stack Height	Drop	Cushioned	Control Elements	Weight	Other
Warne, J. and Warrington, G. [21]	2014	Barefoot-simulated Shod shoes	“Barefoot-simulated” used to refer to minimalist footwear (Vibram Five Fingers KSO) Standard high-quality running shoes of a neutral design (Asics Gel-Nimbus 12)	3.5 mm sole –	10 mm	– Gel	– Plastic element (good torsion and stability).	150 g 400 g	Velcro Laces
Cochrum, R.M., Conners, R.T. and Coons, J.M. [13]	2018	Barefoot Barefoot-style footwear Standard cushioned running shoes	“Barefoot-style footwear” used to refer to “five-toed minimal” footwear, i.e., minimalist.	– – –	– – –	– – –	– – –	– – –	– – –
Lieberman, D., et al. [22]	2010	Barefoot Modern shoes = Shod shoes	Barefoot Asics Gel-Cumulus 10	– –	No 10	No Gel	No Flexible upper. High resistance.	0 335 g	No Laces
Squadrone, R. and Gallozzi, C. [23]	2009	Barefoot Lightweight shoe Neutral Protective running shoe	Barefoot “Lightweight shoe” effectively mimicked the experience of barefoot running (study based on the classical Vibram Five Fingers model).	– 3.5 mm sole –	– 0 –	– – –	– No –	– 148 g 341 g	– Laces around the shoe. –
Rothschild, C. [24]	2012	Barefoot Minimalist running shoes	Vibram Five Fingers.	– –	– –	– –	– –	– –	– –
Fuller, J.T. et al. [16]	2018	Minimalist shoes Conventional shoes	Asics Piraha SP4 Asics Gel Cumulus	– –	5 mm 9 mm	–	– –	138 ± 10 g 333 ± 25 g	Scoring 72% on MI Scoring 12% on MI
Fredericks, W. et al. [25]	2015	Standardised traditional running shoes Minimalist shoe Barefoot without shoes	Nike Air Pegasus 27 Vibram Five Fingers KSO Barefoot	– <5 mm sole –	12 mm 0 mm –	– – –	Neutral. – –	– 150 g –	– – –
Ryan, M. et al. [26]	2013	Neutral shoes Partial minimalist shoes Full minimalist shoes	Neutral shoes were used by all participants who, according to FPI, were pronator, neutral or supinator. Partial minimalist, Nike Free 3.0 v2 Full minimalist, Vibram Five-Finger Bikila.	– – Sole 4 mm	– 4 mm 0	– Yes No	– Incorporated. Flexible upper. No	– 201 g (h) 173 g (m) 188 g	– Laces Lace-tensing system
Sayer, T. et al. [15]	2019	Stability style shoes Neutral style shoes	The stability shoes tested, “Asics Kayano-GS”, obtained a score of 9 on the Footwear Assessment Tool. The neutral shoes tested, “Asics Zacara 3”, had a score of 3 on the same scale.	25 mm 28 mm	13 mm 10 mm	– Moderate cushioning	Heel counter –	260 g 240 g	Laces Neutral. Laces
Langley, B., Cramp, M. and Morrison, S. [27]	2019	Conventional running shoes: motion control, neutral and cushioned.	Motion control: reduce the magnitude and/or rate of pronation. “Asics Gel-Forte” Neutral: combine a number of motion control and cushioning features to provide additional stability. “Asics GT 2000 2” Cushioned: reduce the magnitude and/or rate of impact loading, and increase foot motion. “Asics Gel Cumulus 15”	39 mm 25 mm 26 mm	12 mm 9 mm 11 mm	– “fluid ride” system. Gel cushioning	Control of midfoot pronation by the “guidance line” system. Slight control of movement via the “guidance line” system. “Guidance line” system	377 g 312 g 329 g	Pronator. Laces Slight pronator. Laces Neutral/ Supinators Laces
Knapik, J. et al. [28]	2009	Motion control shoes Stability shoes Cushioned shoes	For low foot arch. For medium foot arch. For high foot arch.	– – –	– – –	– – –	– – –	– – –	– – –
Grier, T. et al. [17]	2016	Traditional running shoes Minimalist running shoes: barefoot style, minimalist and transition shoe.	“Traditional running shoes” differentiated into three subgroups by visual inspection. “Minimalist running shoes” differentiated into three subgroups: “barefoot-style”, “minimalist” and “transition”, according to cushioning and drop (0–9 mm).		12–15 mm 0–9 mm	– –	– –	– –	– –
Kerrigan, J., et al. [29]	2009	Modern-day running shoes = stability running shoes	“Brooks Adrenaline”, chosen for its neutral type and characteristics typical of running shoes.	24 mm	12 mm	Hydroflow	Posting = dual density Lasting = Stroebel board	–	Neutral
Murphy, K., Curry, E. and Matzkin, E. [30]	2013	Modern running shoes = standard running shoes Minimalist running shoes	Description of general characteristics of this type of footwear and of minimalist shoes. “Minimalist footwear”: maintains the freedom and essence of barefoot running.	– –	8–16 mm	Foam or other material. Non-cushioned midsole	– –	– –	– –
Hollander, K. et al. [31]	2015	Barefoot Cushioned minimalist shoe Uncushioned minimalist shoe Standard running shoe	Barefoot Nike Free 3.0 Leguano –	– – 9 mm –	– 4 mm 0 mm –	– Yes – –	– No control of foot arch. – –	– 201 g (h) 73 g (m) – –	– Laces – –
Malisoux, L., et al. [11]	2016	Standard shoes Motion control shoes	– –	– –	10 mm 10 mm	– –	Thermoplastic structure located at the midfoot and, dual-density midsole located at the forefoot.	– –	– –
Pollard, C. et al. [32]	2018	Traditional running shoe Maximal shoes	NB 880 Hoka One One Bondi 4	33.3 mm 41.6 mm	10.1mm 6.9 mm	Conventional cushioning Maximum cushioning	No control of pronation. Rearfoot control. –	305 g –	Neutral. Laces. –
Nigg, B.m. and Segesser, B. [33]	1986	Running shoes	Cushioning, support and guidance. More general use of running footwear.	–	–	–	–	–	–
Anselmo, D.S., et al. [34]	2018	Barefoot, Neutral shoe Motion control shoe	Barefoot Saucony Ideal Saucony Stabil	– – 25.5 mm	– 8 mm 8 mm	– – –	– – Control of severe pronation	– 300 g323 g	– – –
Moody, D. et al. [35]	2018	Traditional footwear Minimalist footwear: 1 Saucony Kinvara 2 Altra the one 3 Vibram El/x Barefoot	Mizuno Wave Rider With low drop, according to the manufacturer. Barefoot	– – – – –	10–12 mm 4 mm 0 mm 0 mm NO	Yes Yes Moderate No No	“Cloud wave” plate to stabilise gait. – – No No	290 g 221 g 230 g 120 g 0	Neutral. Laces. – – – –
Zhang, X. et al. [36]	2018	Neutral shoes Motion control shoes Minimalist shoes Neutral shoe + insole		34 ± 7 mm 37 ± 5 mm 24 ± 6 mm 34 ± 6 mm	11 ± 5 mm 12 ± 2 mm 3 ± 2 mm 9 ± 3 mm	– – – –	Sole stability in sagittal midfoot: minimal to moderate. Sole stability in frontal midfoot: moderate. Sole stability in sagittal midfoot: minimal to moderate/rigid. Sole stability in frontal midfoot: rigid to moderate. No heel control. Sole stability in sagittal and frontal midfoot: moderate to rigid.	303 ± 27 g 332 ± 64 g 222 ± 14 g 301 ± 48 g	– – – –
Da Silva Azevedo, AP., et al. [37]	2016	Conventional shoes Transition shoes Minimalist running shoes	NB 759 NB 890 NB minimus MR10GB	45 mm 40 mm 25 mm	18 mm 12 mm 4 mm	Yes Yes Yes	Inner sole: Etil-Vinil-Acetato. Midsole: Viscoelastic materials. Outsole: Rubber. Midsole: Viscoelastic material, 30% lighter. Outsole: Rubber Midsole: Viscoelastic material, 30% lighter. Outsole: Rubber.	280 g 250 g 209 g	Laces Laces Laces
Roca Dols, A. et al. [38]	2018	Minimalist Boost^®^ EVA Pronation control Air^®^chamber		– – – – –	0 mm 11 mm 9 mm 9 mm 16 mm	No EVA EVA EVA Air chamber	No No No Postero-medial No	172 g 320 g 250 g 286 g 360 g	– – – – –
